# Jasmonic Acid Activates the Fruit-Pedicel Abscission Zone of ‘Thompson Seedless’ Grapes, Especially with Co-Application of 1-Aminocyclopropane-1-carboxylic Acid

**DOI:** 10.3390/plants11091245

**Published:** 2022-05-05

**Authors:** Matthew W. Fidelibus, Peter Petracek, Steven McArtney

**Affiliations:** 1 Department of Viticulture and Enology, University of California, Davis, CA 95616, USA; 2 Valent BioSciences, Libertyville, IL 60048, USA; peter.petracek@valentbiosciences.com (P.P.); steven.mcartney@valentbiosciences.com (S.M.)

**Keywords:** abscission layer, dry drop, fruit quality, harvest aid, plant growth regulators, viticulture

## Abstract

Two studies were conducted to determine how methyl jasmonate (MeJA), jasmonic acid (JA), and 1-aminocyclopropane-1-carboxylic acid (ACC) affect grape berry abscission in the initial days after treatment. The overarching goal was to determine whether JA, with or without ACC, may hold the potential to sufficiently reduce fruit detachment force (FDF) and increase the proportion of berries with dry stem scars while minimizing preharvest abscission, effects that could be useful in the production of stemless table grapes. On Thompson Seedless grapes, JA was at least as effective as MeJA for stimulating berry abscission based on reduced fruit detachment force (FDF) and yielding detached berries with dry stem scars. Further, since previous studies showed that ACC improved MeJA-induced grape abscission, we tested ACC effects on JA activity. We found that JA rapidly induced preharvest berry abscission, confirming previous results. ACC alone did not induce preharvest berry abscission, but ACC improved the effectiveness of JA on reducing FDF and increasing dry stem scar development. These studies also demonstrated that JA-induced abscission occurs within the first day after treatment. Commercial use of JA plus ACC as an abscission agent requires that FDF sufficiently declines, and the incidence of dry stem scars increases, prior to a significant increase in fruit abscission. However, the rapid progression of fruit abscission may require harvest either within 24 and 48 h after treatment or the use of a passive catch system.

## 1. Introduction

The detachment of a grape (*Vitis vinifera* L.) berry from its pedicel generally damages the berry because the vascular tissues and associated parenchyma, collectively known as “the brush”, remain attached to the pedicel and are pulled out of the berry on detachment, leaving an open wound sometimes called a “wet” stem scar on the berry’s stem-end [1,2,3]. Berry detachment may also remove pieces of skin or cause the whole berry to rupture. Such mechanical damage can reduce the yield and quality of machine-harvested grapes for wine [4,5,6] or raisins [3,7]. Stem-end picking damage also limits the quality and storage life of stemless table grapes [1].

**Figure 1:**
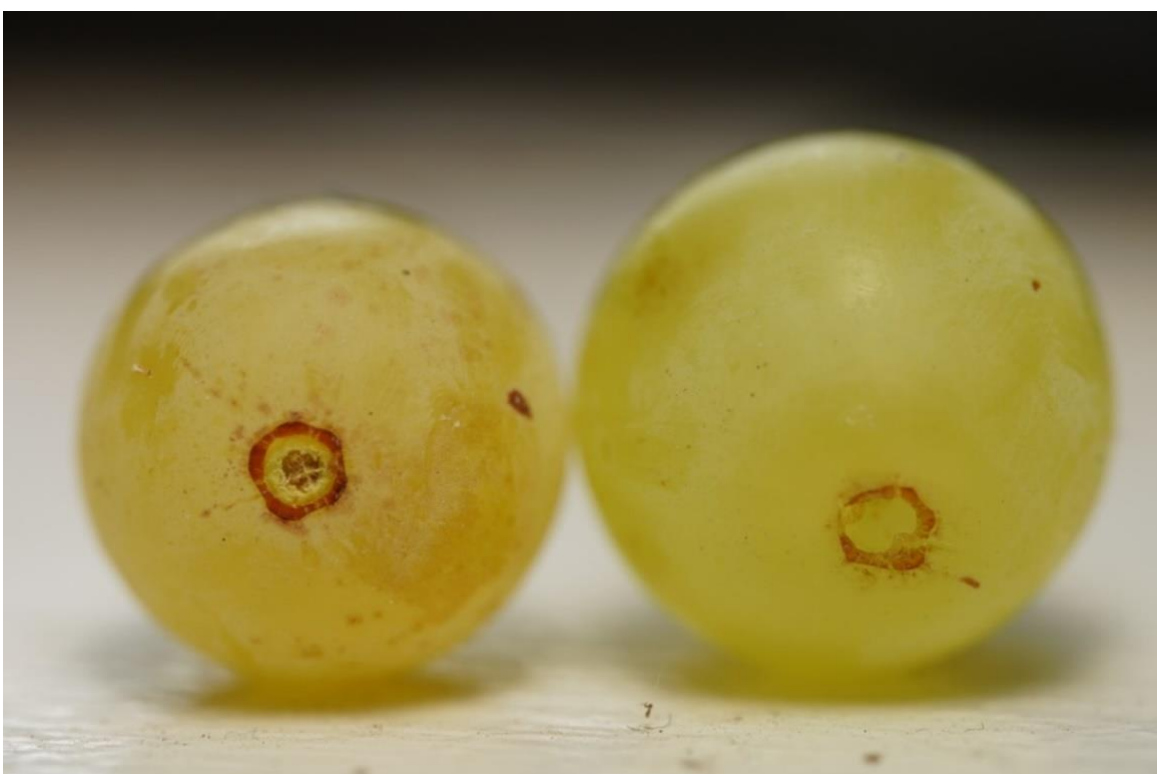
Thompson Seedless grapes treated with an abscission agent develop a dry stem scar (**left**) within a few days after treatment, whereas untreated grapes generally have a wet stem scar after detachment (**right**).

Certain plant growth regulators known as “abscission agents” activate an abscission zone at the pedicel-fruit boundary [8]. The activation of this abscission zone reduces fruit detachment force (FDF) and promotes the development of dry stem scars [2,4,9] (Figure 1). Abscission agents could reduce picking damage and thereby serve as harvest aides if treated grapes can be harvested after the abscission zone is activated, but before the fruit abscises. However, once the abscission zone has been activated, development proceeds quickly and may lead to excessive preharvest fruit drop [10].

The first compound tested as an abscission agent for grape was ethephon [4,11], a phosphonic-acid compound that decomposes to release the gaseous plant hormone ethylene. Ethephon can induce the abscission of mature grape berries within 7 to 14 days after treatment, but high dosages (>1000 ppm) are needed [4,11,12]. The use of ethephon as an abscission agent for grapes would require an application dosage which is higher, and a preharvest interval that is shorter, than those for the current registered use of ethephon on grapes in order to enhance berry color. Such changes could be expected to increase ethephon residues on treated fruit, and it seems unlikely that regulatory agencies would approve a use that could increase ethephon residues on grapes since existing residues are already a concern [13]. However, it should be noted that Ferrara et al. [12] found that effective dosages of ethephon did not result in excessive residues.

Jasmonates, including methyl jasmonate, a natural product, have also been shown to induce the abscission of various fleshy fruits, including blueberry (*Vaccinium corymbosum* L. and *V*. *darrowi* Camp.), orange (*Citrus sinensis* L. [Osb.]), and tomato (*Lycopersicon esculentum* Mill.) [14]. Moreover, the Environmental Protection Agency of the United States ruled that MeJA was exempted from the requirement of a tolerance for residues in or on all food commodities when applied pre-harvest [15], a ruling that could facilitate the development of jasmonates as active ingredients in agrichemicals. A screening trial confirmed that MeJA and coronatine, a jasmonate mimic, also induce abscission in grape [2].

The physiological basis for jasmonate-induced fruit abscission appears to involve ethylene. The exogenous application of jasmonates stimulates ethylene production in several fruits, including apple (*Malus × domestica* Borkh.), orange, grape, strawberry (*Fragaria x* 63 *ananassa* Duch), and tomato [9,16,17,18,19,20,21]. The work carried out on orange [16,17] and grape [9] showed that the application of MeJA stimulated ethylene production by the fruit which was followed by fruit abscission. Malladi et al. [22] provided indirect evidence that MeJA stimulates the abscission of blueberry (*Vaccinium* spp.) fruits at least partly via ethylene action, as the co-application of MeJA with aminoethoxyvinylglycine (AVG), an ethylene biosynthesis inhibitor, attenuated MeJA effects on abscission. However, MeJA still induced some abscission in blueberry, even when co-applied with AVG [22] which suggests that MeJA may initiate some abscission processes independently of ethylene. Moreover, grape berries treated with MeJA or 1-aminocyclopropane-1-carboxylic acid (ACC), the direct precursor of ethylene, produced similar levels of ethylene in the first 2 days after treatment (DAT), but thereafter, berries treated with MeJA produced less ethylene than berries treated with ACC, even though the MeJA-treated grapes generally had lower FDF, greater abscission, and a higher proportion of dry stem scars than the grapes treated with ACC [9]. Together, these findings suggest that ethylene and jasmonic acid can promote fruit abscission via independent pathways and interact to promote abscission.

The potential for synergistic effects has sustained interest in research on the co-application of jasmonates and ethylene-promoting compounds. This is especially important because of the relatively high dosages of MeJA needed for consistent efficacy when applied alone [10] and because MeJA is much more expensive than ethephon. Because of the high rate of ethephon needed for abscission activity, the short time between abscission zone activation and fruit drop, and concerns about ethephon residues, alternatives to ethephon are desired. 1-Aminocyclopropane-1-carboxylic acid is not particularly effective at stimulating abscission on its own, but the co-application of MeJA with ACC improved efficacy in such a way that lower dosages of MeJA could be used [9]. Recent improvements in jasmonic acid (JA) biosynthesis have the potential to make JA and its metabolite MeJA more available and affordable than they are now. The effects of JA on the abscission of fleshy fruits has not been tested, and the relative efficacy of JA versus MeJA with respect to grape berry abscission is unknown. Therefore, two studies were conducted to compare the efficacy of JA and MeJA at inducing abscission of Thompson Seedless grapes and to determine if JA interacts with ACC to promote abscission. 

## 2. Results

### 2.1. Methyl Jasmonate Versus Jasmonic Acid

Methyl jasmonate at 2 mM was ineffective, whereas 4 mM and 8 mM MeJA were equally effective at inducing preharvest abscission, reducing fruit detachment force (FDF), and increasing the proportion of detached berries with dry stem scars (Table 1). Jasmonic acid was as effective or more effective than MeJA at inducing the abscission of Thompson Seedless grapes. Compared with MeJA, JA induced a similar or higher preharvest berry abscission, a similar or lower fruit detachment force, and a similar or higher percentage of detached berries with dry stem scars after treatment with JA, versus MeJA (Table 1). The most effective treatment overall was 4 mM JA, which induced the highest (or among the highest) level of preharvest abscission and percentage of detached berries with dry stem scars (Table 1). Grapes that were treated with 4 mM JA also measured among the lowest FDF values (Table 1).

### 2.2. Effects of Jasmonic Acid, 1-Aminocyclopropane-1-Carboxylic Acid, and Days after Treatment

Without JA, the application of ACC did not induce preharvest berry abscission, and JA and ACC did not interact to affect preharvest abscission (data not shown). With or without ACC, JA rapidly induced preharvest berry abscission, with 14 to 26% abscission by 2 days after treatment (DAT), and 52 to 69% abscission by 3 DAT (Table 2). The effects of JA on abscission were dose-dependent, with 8 mM having the most effect on preharvest abscission at 2 or 3 DAT (Table 2).

In this study, FDF and the proportion of detached berries with a dry stem scar was determined once on 3 DAT. At that time, JA and ACC interacted to decrease fruit detachment force (FDF) and the proportion of berries with a dry stem scar after detachment (Table 3 and Table 4). Without JA, 1000 ppm ACC was needed to decrease FDF (Table 3). With 4 or 8 mM JA, 500 ppm ACC was sufficient to reduce FDF and FDF was not further decreased with additional ACC (Table 3).

ACC increased the proportion of berries with a dry stem scar after detachment without JA, or with 4 mM JA (Table 4). However, most berries treated with 8 mM JA had dry stem scars regardless of ACC and ACC did not further improve the response (Table 4). The co-application of 500 ppm ACC and 4 mM JA was sufficient to maximize dry stem scar development, with no further improvement observed when increasing the amount of ACC or JA (Table 4).

## 3. Discussion

MeJA can stimulate the abscission of many fruits [8] and endogenous JA is known to promote abscission of floral organs [23] and fruits [24], but the data presented here may be the first report of an exogenous application of JA stimulating the abscission of a mature fruit. Moreover, JA appears to be at least as effective, and possibly more effective, than MeJA at stimulating grape berry abscission. Interestingly, this is in contrast with a recent study that showed MeJA was more effective than JA at inducing abscission in lupine flowers [25]. Improved efficacy at lower dosages could facilitate the commercial development of jasmonate-based abscission agents for grapes and other fleshy fruits because these natural products are expensive. In the first study, 4 mM JA was more effective than 8 mM JA; however, the opposite appeared to be the case in the second study. Therefore, additional research is needed to clarify the lowest dosage of JA that is consistently effective. The general range of effective dosages (i.e., 4–8 mM) agrees with previous research that used MeJA as the active ingredient [26].

Abscission zone activation reduces FDF and promotes the development of dry stem scars, both of which could help minimize picking damage and possibly improve the quality of destemmed table grapes. However, the final stage of AZ activation (preharvest fruit drop) is undesirable unless catchment systems can be employed [12]. Previous studies with MeJA suggested that harvest should occur within 3 days after treatment [10,26]. Jasmonic acid also stimulates rapid abscission zone activation, with 14 to 25% abscission observed 2 days after treatment, and 52% to 60% by 3 DAT. Without catchment systems, it may be necessary to harvest treated fruit within 2 days to avoid excess crop loss. This could be logistically difficult, and data are lacking to determine whether the abscission zone would be sufficiently developed by 1 or 2 DAT to provide the potential quality benefits that are desired from abscission agents. Another outcome that needs to be determined is whether the abscission zone could be activated preharvest and develop during postharvest storage. If so, this could be a way to achieve fruit quality benefits for stemless table grapes while minimizing the risk of preharvest fruit drop. 

Lavee [27] demonstrated that preharvest applications of plant growth regulators can affect the postharvest abscission of grapes. Specifically, the preharvest application of 1-naphthaleneacetic acid (NAA) and some other synthetic auxins reduced the postharvest abscission of “Muscat of Alexandria” that were held at room temperature for three days before entering cold storage. However, the abscission of grapes that were placed into cold storage immediately after picking was greatly suppressed, regardless of whether the grapes were pretreated with auxins or not. This suggests that the effect of plant growth regulators on postharvest abscission may depend on postharvest storage conditions. If abscission zone activation and development could both occur postharvest, it may be possible to treat the fruit in a packing house which should enable more efficient application of active ingredients, in addition to preventing crop loss due to preharvest abscission.

The results of the second experiment also demonstrate a benefit in applying ACC with JA, as previously shown with MeJA [9]. The co-application of 500 ppm ACC was sufficient to make 4 mM JA as effective as 8 mM JA in promoting dry stem scars, one of the most important treatment effects with respect to the quality of detached grapes. Because 500 ppm was the lowest dosage of ACC tested, it is unknown if a lower dosage might be as effective, but this should be tested in future research since the 500 ppm and 1000 ppm ACC treatments had similar interaction effects with JA. Likewise, a lower dosage of JA (<4 mM) could be effective, especially if combined with ACC. Thus, additional work should be carried out to determine if combination treatments could enable lower dosages of JA and ACC to be reliably effective.

In conclusion, the exogenous application of JA activates the pedicel-fruit abscission zone of Thompson Seedless grapes, rapidly reducing FDF, increasing the proportion of berries with dry stem scars after detachment, and leading to significant preharvest abscission within 2 days. Treatment effects require more than 2 mM and less than 8 mM JA if applied alone, but possibly less if co-applied with ACC. Additional work is needed to determine if harvest within 2 days after treatment is sufficient to reduce FDF and increase dry stem scar incidence while reducing preharvest abscission. Work should also be carried out to determine if preharvest or postharvest treatments are effective at inducing abscission postharvest.

## 4. Materials and Methods

The experiments were conducted in September 2020 with own-rooted *Vitis vinifera* cv. Thompson Seedless grapevines and supported by an overhead-arbor trellis in a vineyard at the University of California Kearney Agricultural Research and Extension Center, in Parlier, CA. The vines were planted in 1995, trained to quadrilateral-cordons, and cane-pruned, leaving approximately 6 canes per vine, and 15 nodes per cane. Vines were spaced approximately 1.83 m within rows, and 3.65 m between rows which were oriented east to west. All vines were subjected to cultural practices considered normal and ordinary for dry-on-vine raisin grapes in the San Joaquin Valley [28], except that the canes were not severed, and raisins were not made. 

Each of the two experiments used some methods comparable to those employed in a previous study [9]. Clusters on individual vines were considered treatment replicates and each vine was considered a block. Since there were two experiments, each replicated six times, two groups of six adjacent vines were identified, with one group assigned to the first experiment, and the second group to the second experiment.

There were seven treatments in the first experiment: an untreated control; 2 mM, 4 mM, and 8 mM MeJA (Valent BioSciences, Libertyville, IL, USA); and 2 mM, 4 mM, and 8 mM JA (Valent BioSciences, Libertyville, IL, USA). Unique tags were made for each replicate and treatment and placed in different bags, according to their block. Available clusters were randomly assigned to each treatment by pulling the tags out of a bag and tying them to the peduncle of each cluster. The morning after labelling each cluster, solutions were prepared with the proper amount of MeJA or JA in water, with 0.05% (*v*/*v*) of Latron-B1956 spreader-sticker (Loveland Industries Inc., Greeley, CO, USA). Control treatments consisted of water with Latron-B1956. Each solution was placed into a spray bottle (Spritzer, Solo Inc., Newport News, VA, USA), agitated well, and then applied to an appropriately labelled cluster until runoff occurred. Polyethylene shields were used to prevent overspray from contacting other clusters. After the clusters dried, they were enclosed in polypropylene mesh bags to catch any berries at risk of abscission. The bags had a re-sealable flap at the bottom from which abscised berries could be collected and weighed. In the first experiment the berries were collected once, 3 days after treatment. The abscised berries were weighed and discarded, and then each cluster was harvested and taken to a laboratory in their mesh bags where FDF and dry stem scar measurements were made.

At the laboratory, clusters were gently removed from their bags, and small shears were used to sever 10 berries from the top, middle, and bottom part of each cluster, retaining the pedicel and a short section of rachis with each berry. Each berry was then placed in a jig attached to a force gauge (DPS-11; Imada, Northbrook, IL, USA), and force parallel to the fruit axis was applied to the rachis until it detached from the berry and peak force was recorded [10]. After each berry was detached, the stem end was observed and assigned to one of two classes: dry or wet stem scar. The proportion of berries in each sample with a dry stem scar was then calculated. The remaining berries on each cluster were then removed, combined with the ten used for FDF and dry stem scar measurements, and weighed. The total weight of each cluster was determined by adding the weight of any berries that had abscised prior to harvest, and the percentage of preharvest fruit drop was calculated based on the cumulative weight of the abscised berries and the weight of the berries remaining at harvest.

The second experiment had nine treatment combinations: three levels of JA (0, 4, or 8 mM, and three levels of ACC (0, 500, or 1000 ppm) in a factorial design. The ACC was provided by Valent BioSciences (Libertyville, IL, USA). Cluster selection, solution preparation (including Latron-B1956), treatment application, and bagging, were similar to the procedures employed in the first experiment. In the second experiment, cumulative preharvest abscission was determined daily, by weight. After 3 DAT, all the clusters were taken to the laboratory for determination of FDF, dry stem scar development, and cluster weight determination.

All data were subjected to analysis of variance using the GLM procedure of SAS (SAS Inst., Cary, NC, USA). In the first experiment, a randomized complete block design was used, with seven treatments. Means were compared by Duncan’s new multiple range test (*p* < 0.05). In the second experiment, a factorial design was employed with one factor being the dosage of JA, and the second being the dosage of ACC. If JA and ACC did not interact to affect a variable, then only the main effects of JA and ACC were considered. If JA and ACC interacted to affect a variable, then the nature of the interaction was determined, and the data were summarized in two-way tables.

## Figures and Tables

**Table 1:** The effects of different dosages of methyl jasmonate (MeJA) and jasmonic acid (JA) on preharvest berry abscission, fruit detachment force, and the percentage of detached berries with dry stem scars, 3 days after treatment.

Treatment	Preharvest Berry Abscission (%)	Fruit Detachment Force (N)	Detached Berries with Dry Stem Scars (%)
Untreated control	2 c ^z^	1.27 a	3 d
MeJA 2 mM	3 c	1.43 a	13 cd
MeJA 4 mM	39 ab	0.75 b	53 ab
MeJA 8 mM	25 b	0.94 b	28 bcd
JA 2 mM	29 b	1.22 a	29 bcd
JA 4 mM	50 a	0.79 b	60 a
JA 8 mM	24 b	1.18 a	37 abc

^z^ Values are treatment means, *n* = 6. Means followed by a different lowercase letter are significantly different, within columns, according to Duncan’s new multiple range test.

**Table 2:** The main effect of jasmonic acid on cumulative berry abscission through 3 DAT.

Jasmonic Acid (mM)	Cumulative Preharvest Berry Abscission (%)		
	Days after Treatment		
	1	2	3
0	0 ^z^	1 c	15 c
4	0	14 b	52 b
8	0	26 a	69 a

^z^ Values are treatment means, *n* = 6. Means followed by a different lowercase letter are significantly different, within columns, according to Duncan’s new multiple range test.

**Table 3:** The fruit detachment force of grapes treated with different combinations of ACC and JA. Measurements were made 3 DAT.

ACC	Fruit Detachment Force (N)		
	Jasmonic Acid (mM)		
	0	4	8
0	1.37 a^z^ A ^y^	1.25 aA	0.73 aB
500	1.31 aA	0.76 bB	0.41 bC
1000	0.96 bA	0.55 bB	0.41 bB

^z^ Values are treatment means, *n* = 6. Means followed by a different lowercase letter are significantly different, within columns, according to Duncan’s new multiple range test. ^y^ Means followed by a different uppercase letter are significantly different within rows, according to Duncan’s new multiple range test.

**Table 4:** The percentage of grapes with a dry stem scar after detachment. Measurements were made3 DAT.

ACC	Detached Berries with a Dry Stem Scar (%)		
	Jasmonic Acid (mM)		
	0	4	8
0	3 b ^z^ C ^y^	52 bB	79 A
500	10 bB	87 aA	90 A
1000	38 aB	88 aA	87 A

^z^ Values are treatment means, *n* = 6. Means followed by a different lowercase letter are significantly different, within columns, according to Duncan’s new multiple range test. ^y^ Means followed by a different uppercase letter are significantly different within rows, according to Duncan’s new multiple range test.

## Data Availability

The raw data supporting the conclusions will be made available without undue reservation.
